# Immunoglobulin Gene Repertoire Diversification and Selection in the Stomach – From Gastritis to Gastric Lymphomas

**DOI:** 10.3389/fimmu.2014.00264

**Published:** 2014-06-03

**Authors:** Miri Michaeli, Hilla Tabibian-Keissar, Ginette Schiby, Gitit Shahaf, Yishai Pickman, Lena Hazanov, Kinneret Rosenblatt, Deborah K. Dunn-Walters, Iris Barshack, Ramit Mehr

**Affiliations:** ^1^The Mina and Everard Goodman Faculty of Life Sciences, Bar-Ilan University, Ramat Gan, Israel; ^2^Department of Pathology, Sheba Medical Center, Ramat Gan, Israel; ^3^Division of Immunology, Infection, and Inflammatory Diseases, King’s College London School of Medicine, London, UK; ^4^Sackler Faculty of Medicine, Tel Aviv University, Tel Aviv, Israel

**Keywords:** B-cells, gastritis, *H. pylori*, MALT lymphoma, DLBCL, Ig gene, repertoire, somatic hypermutation

## Abstract

Chronic gastritis is characterized by gastric mucosal inflammation due to autoimmune responses or infection, frequently with *Helicobacter pylori*. Gastritis with *H. pylori* background can cause gastric mucosa-associated lymphoid tissue lymphoma (MALT-L), which sometimes further transforms into diffuse large B-cell lymphoma (DLBCL). However, gastric DLBCL can also be initiated *de novo*. The mechanisms underlying transformation into DLBCL are not completely understood. We analyzed immunoglobulin repertoires and clonal trees to investigate whether and how immunoglobulin gene repertoires, clonal diversification, and selection in gastritis, gastric MALT-L, and DLBCL differ from each other and from normal responses. The two gastritis types (positive or negative for *H. pylori*) had similarly diverse repertoires. MALT-L dominant clones (defined as the largest clones in each sample) presented higher diversification and longer mutational histories compared with all other conditions. DLBCL dominant clones displayed lower clonal diversification, suggesting the transforming events are triggered by similar responses in different patients. These results are surprising, as we expected to find similarities between the dominant clones of gastritis and MALT-L and between those of MALT-L and DLBCL.

## Introduction

Chronic gastritis is a common disorder characterized by chronic inflammation of gastric mucosa. In acute gastritis, patients suffer from dyspeptic symptoms including epigastric burning, distention or bloating, belching, episodic nausea, flatulence, and halitosis. In contrast, most patients with chronic gastritis are asymptomatic (1). One of the major causes of gastritis is bacterial infection, most frequently with *Helicobacter pylori* (*H. pylori*). *H. pylori* are Gram-negative bacteria that are present in the gastric mucosa of more than 50% of people and may persist lifelong unless treated (2). *H. pylori* are resistant to elimination by the immune response so the immune system fails to remove the infection effectively (3). Previous studies have shown a strong association between gastritis and *H. pylori* infection, at least in the early stages of gastritis (3, 4). Although rare, organisms other than *H. pylori* (e.g., *Mycobacterium avium*-intracellulare, Herpes simplex, Cytomegalovirus, and Epstein–Barr virus) can invade the gastric mucosa and cause inflammation (5, 6). Gastritis can also be initiated *de novo*, as an autoimmune disease (7). In either case, prolonged antigenic stimulation causing chronic inflammation might further contribute to the development of some malignancies (8), such as gastric mucosa-associated lymphoid tissue (MALT) lymphoma (9–16).

Mucosa-associated lymphoid tissue lymphoma (MALT-L) is a low-grade B-cell lymphoma. It grows slowly and remains confined to one organ for a relatively long time. Stomach MALT-L exemplifies the close link between chronic inflammation and lymphomagenesis. B-cells of MALT-L are related to normal marginal zone cells. Their IgH variable region gene sequences exhibit features of post germinal center B-cells, such as somatic hypermutation (SHM), implying that the clone has expanded in the presence of an antigen (17). MALT-L is often associated with bacterial infection, most commonly by *H. pylori* bacterium (7–9, 15–17).

A possible outcome of low-grade B-cell lymphomas such as MALT-L is the transformation into a more aggressive lymphoma such as diffuse large B-cell lymphoma (DLBCL) (18, 19). Gastric DLBCL is a fast-growing, aggressive B-cell malignancy characterized by diffuse proliferation of large neoplastic lymphoid B-cells (20, 21). DLBCL is known to represent a heterogeneous group of malignancies, comprising either germinal center-like cells exhibiting intra-clonal diversity or “activated B-cell-like” cells, which do not (22, 23).

During the clonal expansion of B-cells in response to an antigen, Ig gene sequences from clonally related B-cells (i.e., B-cells that are derivatives of the same B-cell ancestor) accumulate mutations via SHM and thus diversify. Clonally related cells are identified by identical V(D)J segments and by highly homologous sequences of the complementary determining region (CDR) 3 of their Ig genes. An easy way to track and analyze the relationships between clonally related Ig gene sequences is by using lineage trees. The tree root is the ancestor sequence, usually the rearranged, pre-mutation sequence. Each tree node represents a single mutation (point mutation, insertion, or deletion). Lineage trees have been used in order to quantify the differences between the dynamics of SHM and antigen-driven selection in different lymphoid tissues, species, and disease situations. Our lineage trees-based mutation analysis has demonstrated its usefulness in previous studies of aging (24), autoimmunity (25–28), and chronic inflammation (29). Recent work on B-cell malignancies done in our lab (30–32) showed differences in tree properties between lymphomas and controls. Lymphoma trees were more branched and had longer trunks compared to controls, indicating a higher intra-clonal diversification and a longer mutational history. Intra-clonal diversification was also shown in chronic lymphocytic leukemia cases (33–35), in marginal zone lymphoma cases (36, 37) and in intestinal DLBCL cases (21). In addition, lymphoma and controls exhibited similar mutation rates and same SHM motifs. Follicular lymphoma (FL), which is considered a less aggressive lymphoma, displayed higher diversity than DLBCL and highest recent diversification events, suggesting that the more aggressive lymphoma diversifies the least (38–40).

In the present study, we used repertoire, lineage tree, and mutation analyses to investigate whether and how B-cell repertoires, clonal diversification, and selection mechanisms in gastritis, gastric MALT-L, and DLBCL differ from each other and from normal responses. The two types of gastritis (positive or negative for *H. pylori*) were found to have similar repertoires and diversification. MALT-L clones were found to be more diversified and had longer mutational histories compared with all other conditions, but the dominant clones of MALT-L (defined as the largest clones in each sample) were different from those of all other conditions. DLBCL dominant clones, however, displayed lower diversification. These results are surprising, as we expected to find similarities between the dominant clones of gastritis and MALT-L and between those of MALT-L and DLBCL, according to the hypothesis that these are often sequential steps of inflammation and transformation.

## Results

### Repertoires in gastritis with *H. pylori* background were as diverse as those in gastritis negative for *H. pylori*, and contained similar V(D)J combinations

**Figure 1:**
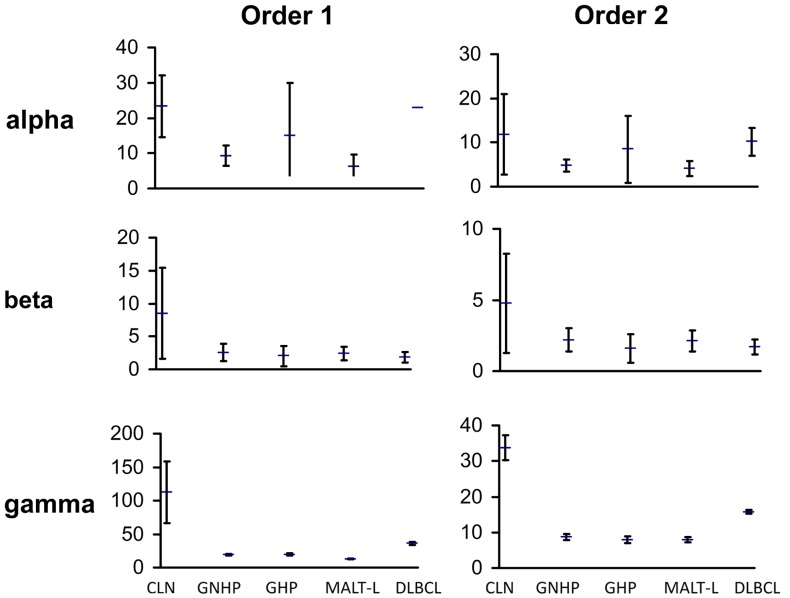
**Graphic presentation of alpha, beta, and gamma diversity indices of order 1 and 2**. Diversity measures were calculated, as described in the Section “Materials and Methods,” based on the Shannon entropy and the Simpson concentration diversity indices. The alpha diversity measure represents the average sample diversity in each condition/population. The gamma diversity measure represents the “global” repertoire diversity across all samples studied in each condition/population. The beta diversity measure represents the diversity component resulting from the variability between samples. In our Ig gene repertoire studies, the abundance data (numbers of unique sequences) of antibody clones in each sample was used to estimate the mean, standard error, and 95% confidence intervals (CI) of the total number of unique sequences in clones within each sample. The CI allows us to compare between diversity indices of different conditions. The error bars show the standard errors.

**Figure 2:**
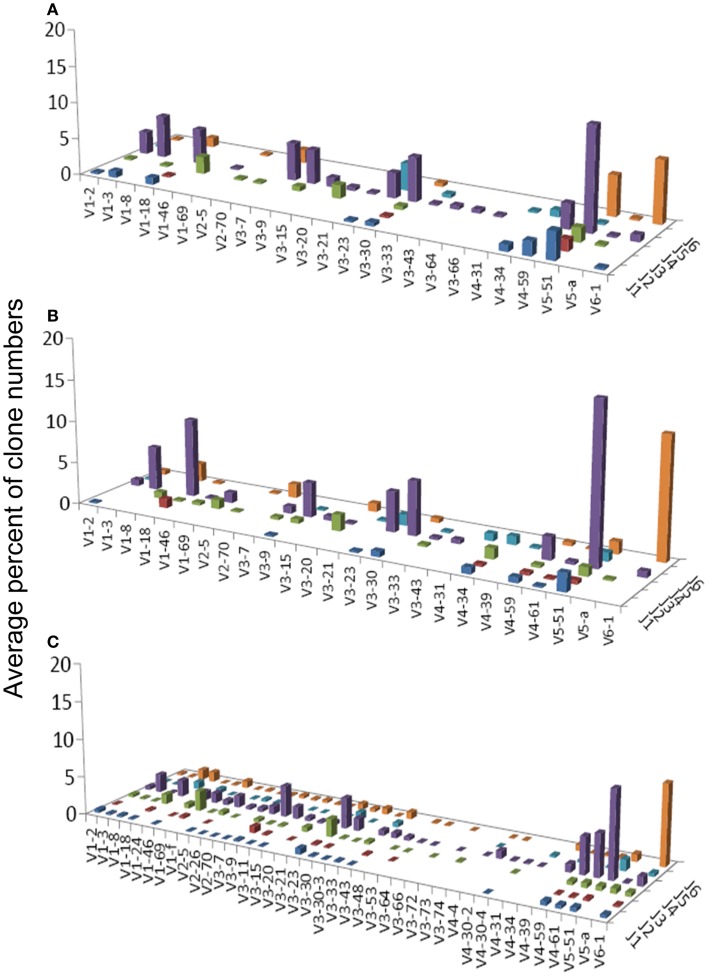
**Average percentages of clones in each VH–JH combination, in (A) GHP, (B) GNHP, and (C) CLN samples**.

We compared the repertoires in both types of gastritis, with *H. pylori* background (GHP) or without *H. pylori* background (GNHP), and examined the differences between them. We expected the repertoire in GHP to be less diverse due to the response to the bacterium, as previous studies showed that monoclonality is frequently found in GHP samples [(41–43) and others]. In contrast to our expectation, the confidence intervals (CI) of alpha, beta, and gamma diversity indices of both orders were overlapping (Figure 1), implying the average individual biopsy diversities, the variability of diversities between individual biopsies, and the overall pool diversities in GHP and GNHP were not statistically different. Indeed, most V(D)J combinations observed were expressed in both gastritis types (Figures 2A,B).

Gastritis with *H. pylori* background and GNHP were the most similar conditions (similarity index of 0.543, Table 1), although one GHP sample (the second GHP sample in Table S1 in Supplementary Material) had an extremely high alpha diversity index compared to the other two samples (data not shown). This contradicts our expectation of narrower repertoires in GHP samples due to the presence of *H. pylori*. However, if the one highly diverse GHP sample is excluded from the analysis, the confidence interval of alpha of GHP becomes narrower (3.9–11.25), and lower than that of GNHP. It is possible that the highly diverse sample reflected additional ongoing responses.

**Table 1:** **The average similarity between each pair of conditions**.

	CLN	GNHP	GHP
CLN	0.211	0.295	0.343
GNHP		0.408	0.543
GHP			0.478

*Similarity measures were calculated between all clones in all samples in the compared conditions. In lymphomas, only the dominant clone(s) are relevant, as the rest of the clones in each sample represent other B-cells present in the tissue, which are not related to the malignancy. Thus, MALT-L and DLBCL are not included in the calculation of similarity measures because the dominant clones in these conditions cannot be compared to the full repertoire samples from other conditions*.

VH1-3/JH4 was a common combination in both GNHP and GHP VH–JH repertoires, but not so prominent in repertoires of other conditions (Figures 2A,B). These combinations contained several DH genes in both GNHP and GHP. However, identification of D genes should be taken with caution, as SoDA always finds a D gene, even when this is based on too-few nucleotides to be reliable. Table 2 summarizes all common combinations and genes found in our study and their relationships with other clinical conditions as implicated in the literature.

**Table 2:** **A summary of frequent combinations and genes in conditions from our study and from other studies**.

Gene or combination	Common in our study in	Appeared in the literature in relation to
VH1-2	MALT-L^a^	Self-reactive antibodies, Bahler et al. (44)
		Chronic lymphocytic leukemia (B-CLL), primary central nervous system lymphomas, and splenic marginal zone lymphomas, Walsh and Rosenquist (45)
VH1-3	GNHP, GHP	B-CLL, Fais et al. (46)
VH1-18	GNHP, GHP, DLBCL	B-CLL, Fais et al. (46)
		Autoreactive gene, Yamashita et al. (47)
		BM-DLBCL, gastric MALT-Ls, Bende et al. (48)
VH1-8	DLBCL	B-CLL, Pimentel et al. (49)
VH1-69	MALT-L^a^, DLBCL	Rheumatoid factor, Bende et al. (48), Matsuda et al. (50)
		Gastric MALT-Ls, Bende et al. (48)
		B-CLL, Fais et al. (46), Pimentel et al. (49), Johnson et al. (51)
VH2-26/JH5	MALT-L^a^	FL, Bayerl et al. (52)
VH2-26	MALT-L^a^	B-CLL, Pimentel et al. (49)
		Hairy cell leukemia, Hashimoto et al. (53)
VH3-7	MALT-L Dominant, DLBCL	Rheumatoid factor, Bende et al. (48), Matsuda et al. (50)
		Rheumatoid arthritis, Nakamura-Kikuoka et al. (54)
		Sjögren syndrome, Bahler and Swerdlow (55)
		B-CLL, Fais et al. (46)
		BM-DLBCL, Yamashita et al. (47)
		Gastric MALT-Ls, Bende et al. (48)
VH3-23	DLBCL	IgM+ B-cells, Brezinschek et al. (56)
		Naïve B-cells, Wu et al. (57)
		Anti-DNA auto-antibodies, Matsuda et al. (50)
		Hepatitis C virus-related mixed cryoglobulinemia, Perotti et al. (58)
		Unmutated VH3-23 in transformation from B-CLL into DLBCL, Mao et al. (59)
		Gastric MALT lymphomagenesis, Sakuma et al. (60), Lenze et al. (61), Alpen et al. (62), Siakantaris et al. (63)
		BM-DLBCL, Yamashita et al. (47)
		B-CLL, Pimentel et al. (49)
VH3-30	DLBCL	Rheumatoid factor, Bende et al. (48), Matsuda et al. (50)
		Gastric MALT lymphomagenesis, Sakuma et al. (60), Lenze et al. (61), Alpen et al. (62), Siakantaris et al. (63)
		B-CLL, Pimentel et al. (49)
VH3-30/JH4	DLBCL	FL, Bayerl et al. (52)
VH5-51 and VH6-1	CLN, GNHP, GHP, and DLBCL	Auto-antigens, Matsuda et al. (50)
	Dominant	

*The dominant segments are marked with “Dominant” indication. The dominant segments/combinations are those that appeared in the largest clone in each sample*.

*^a^Represents frequent genes or combinations in the repertoire of unique sequences*.

In the case of GNHP, the DH3 and DH6 families were found to be preferred, as combinations of V6D3J6 and V6D6J6 were used significantly more than expected (Table 3; Figure 2B). Other over-expressed DH genes were used in less prominent combinations in the observed repertoire. Other combinations used in GNHP and GHP, such as VH3-7, VH3-23, and VH3-30 – all with JH4 – used several DH genes from different DH families.

**Table 3:** **VDJ combinations that were over-expressed in each condition^a^**.

Condition	Combination	*p*-Value	Mean difference^b^
GNHP	V2D1J6	0.009	3.57
	V3D5J4	0.002	4.38
	V3D0J5	0.001	7.84
	V6D6J6	0.035	3.84
	V6D3J6	0.042	3.60
GHP	V3D0J5	0.035	10.44
	V5D3J2	0.015	3.26
DLBCL	V1D1J6	0.011	1.30
	Dominant^c^	
	V1D4J3	0.000	4.94
	V4D6J4	0.046	2.70
	V5D1J4	0.001	1.80
	Dominant^d^	
	V5D7J4	0.036	2.20
	V6D6J6	0.000	3.47
	V6D3J6	0.003	3.50

*^a^There were no VDJ combinations that where over-expressed in MALT-L samples, thus MALT-L does not appear in the table*.

*^b^Represents the value of log_2_(observed/expected)*.

*^c^This combination was found in DLBCL samples number 1, 2, 3 (sample numbers according to Table S1 in Supplementary Material). The dominant combinations are those that appeared in the largest clone in each sample*.

*^d^This combination was found in DLBCL sample number 5*.

Gastritis without *H. pylori* background and GHP presented almost identical gene usage patterns, having VH5-51/JH4 and VH6-1/JH6 as the two most frequent combinations (Figures 2A,B). VH5-51 and VH6-1 have been shown to often participate in earlier stages of repertoire development via positive selection by auto-antigens (Table 2). These two combinations also appeared in our control lymph node (CLN) samples (Figure 2C), so they probably have no specific connection to gastritis or *H. pylori* response. However, the VH1-18/JH4 combination was more frequently used in GNHP than in GHP, and was not prominently observed in other conditions. As VH1-18 is an autoreactive gene and was found in several gastric MALT-Ls (Table 2), VH1-18 may be involved in the development of gastritis regardless of the presence of *H. pylori*.

**Figure 3:**
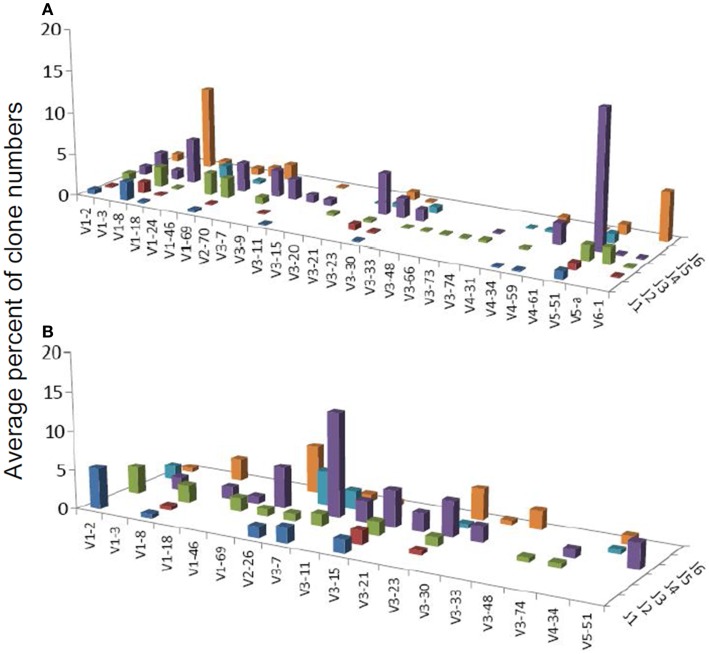
**Average percentages of clones in each VH–JH combination, in (A) DLBCL and (B) MALT-L samples**.

One combination was over-expressed in both types of gastritis (V3D0J5), and two combinations (V6D3J6 and V6D6J6, Table 3) were over-expressed in GNHP (as dominant clones) and in DLBCL samples (not the dominant clones). As can be seen in Figures 2B,C and 3A, many sequences used the V6–J6 combination in GNHP and DLBCL, but also in the controls. Thus, this combination is very frequent in immune responses, and cannot be ascribed to a specific condition. However, the combination V3D0J5 may represent an antibody that is effective in the gastric environment, related to inflammatory processes, or participates in both.

To conclude, both types of gastritis presented similar repertoires and diversity properties, in contrast to our expectation.

### Gastric MALT-L exhibited unique V(D)J combinations

Several studies have demonstrated that gastric MALT-L is often associated with a bacterial infection, most commonly by *H. pylori*; another association has been revealed between gastritis and gastric MALT-L (11–13). Therefore, we expected to find similar V(D)J combinations when comparing the two conditions. Surprisingly, the dominant MALT-L V(D)J combinations were very different from those in GHP. While GHP showed an extensive use of JH4 family genes and several common combinations, of which the most frequent was VH5-51/JH4, in MALT-L dominant clones were VH3-7/JH4, VH1-69/JH6, and VH1-2/JH1. VH3-7 is frequently found in rheumatoid factors and was selectively expressed by patients with rheumatoid arthritis and Sjögren syndrome. Preferential use of these genes and combinations has been reported in several types of lymphomas and leukemias (Table 2).

Table S2 in Supplementary Material presents the combinations that were over-expressed in one condition while under-represented in the other. It can be seen (from the “Mean deviation” column) that over-expressed combinations were almost absolutely from either MALT-L or DLBCL samples (dominant clones only). As DLBCL contained dominant combinations that also appeared in other conditions, this supports the observation of different dominant combinations in MALT-L compared to those observed in other conditions. These combinations may relate to the malignancy, but this remains to be explored.

We also compared the dominant clones in DLBCL and MALT-L samples, as in some cases DLBCL appears in association with MALT-L (18, 19). DLBCL is considered in these cases to result from clonal transformation of large cells within the low-grade lymphoma (64, 65). Hence, we expected to identify similar segment combinations on the dominant clones from the two conditions. However, the dominant clones of MALT-L samples were different from those of DLBCL (Figures 3A,B). As mentioned above, the dominant clones in MALT-Ls were VH3-7/JH4, VH1-69/JH6, and VH1-2/JH1, while in DLBCLs these combinations were found, but were not the dominant clones. VH5-51/JH4 and VH6-1/JH6 were frequent combinations in all conditions, except in MALT-L, suggesting they may have some advantage in binding common antigens. In terms of unique sequences, MALT-Ls presented completely different dominant combinations from DLBCL (VH1-2/JH1, VH1-69/JH6, VH2-26/JH5, and VH3-7/JH4, data not shown). Preferential use of these genes and combinations has been reported in several types of lymphomas and leukemias (Table 2). In addition, VH3-7/JH4, which was the most frequent combination in MALT-L dominant clones, appeared in all other conditions but with dramatically lower numbers. As mentioned above, VH3-7 participates in the formation of auto-antibodies and was found in several gastric MALT-Ls. Some MALT-Ls were found to use VH genes previously associated with auto-reactivity. This suggests that B-cells in MALT-L react with self-antigens (66), different from those that arouse in GHP and DLBCL responses.

### MALT-L dominant clones had longer diversification history, in contrast to DLBCL clones

**Figure 4:**
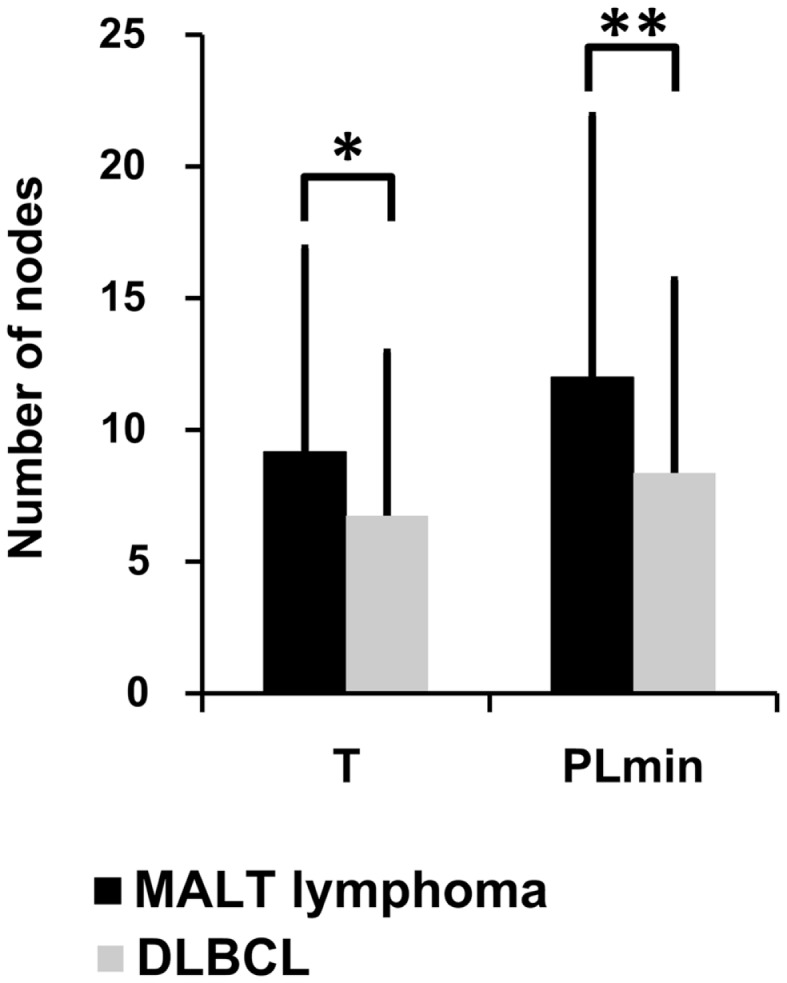
**Lineage tree analysis – comparison between dominant clones from the three MALT-L samples (24 trees) and the five DLBCL samples (47 trees)**. There was more than one tree per sample, as we included all clones with the same VH and JH genes (but different alleles) in the dominant clone in each sample, because they might be related to the dominant clone and falsely attributed to other alleles. Significant differences were found in trunk length (T) and minimal path length (PLmin). An asterisk (*) represents *p*-value <0.01; two asterisks (**) represents *p*-value <0.005.

Lineage trees of the MALT-L dominant clones had significantly longer trunks (T) and path lengths (PLmin), which are tree length measures, than all other conditions (Figure 4; Figure S1 in Supplementary Material). In addition, according to the correlation of tree properties with the dynamic parameters of the secondary B-cell response (67), longer trunks correlate with a lower initial affinity, and longer paths also correlate with a lower selection threshold. This suggests that diversification history in MALT-L dominant clones was longer than that of other conditions.

On the contrary, DLBCL dominant clones had significantly shorter trunks and path lengths than those of GHP (and MALT-L), and in general, the lowest tree length measures. Dominant clones of DLBCL presented similar tree length measures to those of CLN (Figure 4; Figure S1 in Supplementary Material). This is in line with the above-described observation of similarity between DLBCL and CLN. The shorter lengths observed in DLBCL, which correlate with high initial affinity and selection threshold, may indicate a shorter diversification process compared to MALT-L and GHP (21). A possible explanation for this is that, because MALT-L is an indolent lymphoma and DLBCL is an aggressive lymphoma, the latter usually has less time to diversify until it is discovered and treated. Figures S2 and S3 in Supplementary Material show representative examples for MALT-L and DLBCL dominant trees, respectively.

The fact that MALT-L dominant clones had larger trunks, path lengths, and distance from the root to any split node, thus probably lower initial affinity than DLBCL dominant clones, suggests that DLBCL dominant clones started as responses to specific (yet-unknown) antigens, with probably higher initial affinity than the responses that initiated MALT-Ls. That is, high affinity and vigorous response may be risk factors for aggressive lymphoma development. In terms of selection, these results show that selection thresholds in MALT-L dominant clones were the lowest among all other conditions. Low selection pressure may simply be the result of abundance of antigen, and this may indeed be the case in gastric MALT-Ls.

Dominant clones from the two types of gastritis presented similar tree length measures, which correlate with the observed similar repertoires.

## Discussion

In this study, we investigated the relationships between four related conditions of the stomach: gastritis positive or negative for *H. pylori*, gastric MALT-L, and gastric DLBCL. As previous studies showed, these conditions sometimes appear successively, as prolonged stimulation during chronic gastritis may result in the development of gastric MALT-L, which in some cases further transforms into DLBCL. We examined the clonal repertoires of the IgH variable region genes (or in the case of lymphomas, the dominant clones, which are defined as the largest clone in each sample) and the lineage tree characteristics in each condition, in order to find similarities or differences between these conditions.

Both types of gastritis presented similar IgH variable region gene repertoires and lineage tree characteristics, in contrast to our pre-study assumptions. However, although the GNHP biopsies were negative for *H. pylori*, it could be present in the tissue in undetectable amounts, and thus affect the repertoire of the B-cells in its surroundings. Moreover, both types of gastritis used the VH1-18 gene, which may be involved in the development of gastritis regardless of the presence of *H. pylori*; this remains to be elucidated, as this was not the dominant combination in both type of gastritis. We expected the repertoire in GHP to be less diverse than that of GNHP due to the response to the bacterium, which is expected to elicit only specific clones. In contrast to our expectations, GHP samples showed at least as diverse repertoires as GNHP (Figures 1 and 2A,B). An explanation for a high diversity in GHP might be the phase variation of *H. pylori*, which is the generation of intra-strain diversity that is important for bacterial niche adaptation (68), and could cause variability not only in the bacterial strains but also in the responding antibody repertoire. Hussell et al. (69, 70) showed that the extreme variability of *H. pylori* strains led to diverse T cell responses. Moreover, they showed that B-cells did not respond to *H. pylori* themselves, but required contact-dependent help from *H. pylori*-specific T cells, and their Ig genes responded to auto-antigens, similar to our observations. Alternatively, the repertoires in the biopsies – even GHP biopsies – may reflect immune responses to a variety of pathogens, including but not limited to *H. pylori*.

Although in many cases DLBCL is associated with MALT-L, the two types of lymphomas presented different dominant clone combinations and lineage tree characteristics. DLBCL may develop after prolonged stimulation during gastritis, derive from a low-grade malignant clone, or it can initiate *de novo*, depending on the mutations in each clone. In this study, as MALT-L and DLBCL presented different dominant clone combinations, in contrast to our expectations; we speculate that in these cases DLBCL may have initiated *de novo*. MALT-L samples presented different lineage tree characteristics from those of all other conditions, although we expected MALT-L to resemble GHP. In fact, we identified preferential use of the autoreactive gene VH3-7 in MALT-L samples. VH3-7 was one of the common VH genes in GHP (but not the dominant clone). These findings suggest that gastric MALT-L is derived from highly restricted B-cell subsets probably resulting from specific antigenic stimulation, such as with *H. pylori* (15). It is possible that B-cells in MALT-L react with self-antigens (66), however, the role of self-antigens in the development of the malignancy has yet to be examined. Moreover, lineage tree drawings demonstrated longer trunks and path lengths in MALT-L dominant clones, compared with all other conditions. These differences in tree characteristics correlate with lower initial affinity and lower selection threshold, respectively. Low selection pressure may simply be the result of abundance of antigen, and this may indeed be the case in gastric MALT-L. The above may indicate that MALT-L has undergone a longer mutational history than other conditions. On the contrary, the shorter lengths observed in DLBCL dominant clones may be a result of shorter diversification and responses to specific (yet-unknown) antigens, with higher initial affinity compared to MALT-L and the two types of gastritis (21). The latter may be risk factors for aggressive lymphoma development.

We observed some similar VH–JH combinations in all conditions, together with over-expressed and preferred combinations unique to gastritis, MALT-L, and DLBCL samples. These combinations should be investigated in order to further understand their role in the development of each condition. For example, the relatively extensive use of combinations, which were previously found in other malignancies, in DLBCL samples in this study reinforces the notion that some DLBCL clones had developed from MALT-L clones. Moreover, the fact that the dominant combinations were identical in all conditions except in MALT-L (and that MALT-L did not contain this combination at all) is interesting and should be further investigated. We also identified frequent VH genes in GHP repertoires and in the dominant clones in MALT-L samples, which were also found in autoimmune diseases. It was previously shown that some autoreactive B and T cells are activated during *H. pylori* infection (71). The connection between the appearances of these VH genes in GHP and MALT-L samples from our study and in autoimmune diseases remains to be explored. There was no prominent trend toward any V, D, or J gene family in the over-expressed combinations in each of the conditions. However, combinations that were over-expressed in lymphoma dominant clones compared to other conditions had a clear preference toward the use of VH2, JH1 in MALT-L and VH1, JH2 in DLBCL. The role of these combinations and gene families in lymphomas is still unknown.

It should be noted that, because we studied formalin-fixed paraffin-embedded archival biopsies, we had access to only a limited number of biopsies, and limited amounts of DNA, as DNA in the preserved biopsies is often denatured (72). More samples in more conditions will have to be studied in order to give a clearer picture of the roles of specific V(D)J genes and combinations in inflammation and malignancies. Moreover, the similarity between conditions is also affected by the limited number of samples in each condition. This may cause the similarity calculations to be biased toward random features of the samples that may characterize a certain condition, and thus affect the interpretations. However, the similarity between samples within each condition was not very large, so it is unlikely that some random feature is common to all samples of the same condition. Coincident with obtaining more samples, we intend to use the Illumina high-throughput sequencing (HTS) in future studies, in order to avoid the 454 sequencing artifacts, which resulted in discarding many sequences (73). A possible argument could be raised regarding the exclusion of duplicate sequences from our analysis. As mentioned in the Section “Materials and Methods,” duplicate sequences may stem from the PCR amplification, and may cause misidentification of dominant clones. Although we stringently defined duplicate sequences as those that had the exact nucleotide composition and equal lengths, most of the sequences differed not only by their lengths, but also in the mutations they include. Table S5 in Supplementary Material summarizes the distributions of the number of sequences in the tree-nodes in the dominant and second dominant clones in lymphoma samples. As can be seen from Table S5 in Supplementary Material, most of the nodes contained only one sequence, implying that most of the sequences differed not only in their lengths, but also in the mutations they include. There were, however, several nodes that contained more than one sequence. This does not necessarily indicate that those sequences are duplicates, as the clone-tree is built according to the aligned sub-sequences that are shared between all sequences in the clone. This means that sequences included in a specific node could differ in their ends, and not necessarily be duplicates.

All conditions in this study presented similar mutation rates and the same SHM targeting motifs. Replacement and silent mutation analysis revealed a strong selection against replacement mutations in the CDR regions of all conditions. This may indicate that most of the examined IgH variable region gene sequences represented B-cell receptors (BCRs) that were already highly specific to their antigens and thus selection operated against replacement mutations in their CDR regions, which are responsible for antigen binding. These results are also consistent with previous work on lymphomas from our lab (30).

In this study, we used the Morisita similarity index in order to measure similarity between conditions (as mentioned in the Section “Materials and Methods”). Similarity measures were calculated between all clones in all samples in the compared conditions. In lymphomas, only the dominant clone(s) are relevant, as the rest of the clones in each sample represent other B-cells that are also present in the tissue, but are not related to the malignancy. Thus, the MALT-L and DLBCL conditions were not included in calculation of similarity measures exactly because the dominant clones in these conditions cannot be compared to the whole other samples in other conditions. In addition, Morisita similarity index is of order 2, which emphasizes large clones. This affects the similarity results. However, we are interested in the larger clones in each condition as they probably represent the dominant responses.

In summary, we showed that gastritis positive or negative for *H. pylori* presented very similar IgH variable region gene repertoires. This suggests that the diverse stomach repertories do not change much due to the presence of the bacteria, and moreover, GHP does not become oligoclonal (or at least with narrower repertoire) due to *H. pylori*. MALT-L, however, presented different and unique dominant clone gene combinations, which can result from specific antigenic stimulation. As was mentioned in the Section “Introduction,” several studies showed that *H. pylori* causes gastritis, and suggested that prolonged gastritis can lead to MALT-L, and that prolonged MALT-L can develop into DLBCL. This flow and graduation of diseases led us to the assumption that the repertoires (VDJ combinations) in these conditions would be similar, because these conditions were initiated by the bacteria, and several clones got out of control to progress into lymphoma. Moreover, the diversity in these conditions was expected to be narrower than that in CLN, and to be progressively lower as the conditions proceed toward the aggressive lymphoma. However, the results differed from what we expected. In addition, some combinations did appear in several conditions, but not in MALT-L, and the DLBCL dominant clones also appear in other conditions (so they were not unique to the cancer). We speculate that the transformation into MALT-L, after the prolonged stimulation by the chronic GHP, amplified specific combination(s) that were also found in GHP but in a lower frequency (such as VH3-7). The two types of lymphomas differed in their dominant clone gene combinations and lineage tree characteristics, suggesting differences in the abundance of antigens, if not in their nature, which remain to be explored.

## Materials and Methods

### Histopathological specimens

Five gastric DLBCL biopsies, 3 gastric MALT-L biopsies, 10 chronic gastritis biopsies (3 with *H. pylori* background and 7 that were negative for *H. pylori*), and 19 reactive lymph node biopsies (which served as controls), each from a different patient, were selected from the pathology department archives in the Sheba Medical Center (Table S1 in Supplementary Material). Tissue biopsies were taken during resection procedures and were used in this study in accordance with institutional Helsinki committee guidelines and approval. Histochemical stains, by Hematoxylin-Eosin (H&E) and Giemsa, were performed for histological evaluation and *H. pylori* identification. For diagnosis of lymphoproliferative disease and characterization of lymphocyte populations, immunohistochemical stains (e.g., CD20, CD3, CD23, CD21, cyclin-D1, Ki67, and IgD) were also performed. All cases were revised by two independent pathologists to confirm the diagnosis.

### DNA extraction

Paraffin-embedded blocks were cut using a microtome to get extremely thin slices of tissues (sections). Each of the biopsies was consecutively cut to yield 10–20 sections of 4 μ each, depending on tissue size. All sections from each biopsy were inserted into an eppendorf tube with 200 μl water (Sigma) and were heated in 90°C until the paraffin was melted. After the tubes were centrifuged at full speed (14000 rpm) for 1 min, a paraffin ring was created and could easily be removed from each of the tubes. Water was drawn from the tubes while tissues remained in the tubes. Extraction of DNA was then performed using the QIAamp DNA Mini Kit (or the QIAamp DNA Micro Kit for very small samples) according to the QIAGEN protocol.

In some cases, a micro-dissection was needed. First, H&E stained thin sections were reviewed by a pathologist, and areas of interest were outlined. The tissues were cut from 5 to 10 sections that were placed onto five slides per tissue. The slides were heated in 90°C for 15 min. Next, the slides were hydrated (5 min soaking in Xylene, brief immersion in Ethanol 100, 96, and 70% in this order). The slides were then placed to dry. According to the outlined stained slides of each tissue, the hydrated slides were scratched with buffer ATL (QIAamp DNA Mini Kit, QIAGEN) and sample scrapings were picked up into eppendorf tubes. Extraction of DNA was then performed using the QIAamp DNA Mini Kit (or the QIAamp DNA Micro Kit for very small samples) according to the QIAGEN protocol.

### PCR amplification and high-throughput sequencing

For each sample taken from each biopsy, semi-nested PCR was performed using the same forward primers for the two PCR rounds and two different reverse primers as described below.

Forward primers from FR2 region:

VH1: 5′-TGCGMCAGGCCCCYGGACAAR-3′,
VH2: 5′-ARGRAAGGCCCTGGAGTGG-3′,
VH3: 5′-CCAGGCTCCAGGSAAG-3′,
VH4: 5′-MGGGAAGGGRCTGGAGTGG-3′,
VH5: 5′-GAAAGGCCTGGAGTGGATGGG-3′,
VH6: 5′-TTGAGTGGCTGGGRAGGAC-3′.

Reverse primers:

First round – JH1R: 5′-TGAGGAGACGGTGACCAGGGT-3′,
Second round – JH2R: 5′-TGACCRKGGTHCCYTGGCCC-3′.

There is no specific primer for VH7 gene family in the FR2 region, as the VH7 primer in this region is very similar to that of VH1, thus, it amplifies the VH1 family and creates a very strong VH1 bias.

The primers were augmented for HTS experiments by the addition of 5′ sequencing adapter elements and 10-nucleotide unique sample molecular identification (MID) tags according to the 454 FLX Titanium chemistry protocol (Roche) (74). Proofreading Taq DNA polymerase (ABgene) was used in PCR reactions according to the manufacturer’s protocol. PCR reaction was performed on a sample of 50 ng DNA from each sample, with slight changes according to calibration (because DNA was taken from different tissues, and each tissue can differ in the percentages of lymphocytes). PCR products were separated on a 2% agarose gel stained by ethidium bromide. Clear bands were cut from the gel and DNA was extracted using the MinElute Gel Extraction kit (QIAGEN), according to the manufacturer’s protocol. Sequencing of small samples of the PCR products by the classic Sanger method (after cloning to pGEM – T-easy vector) was performed in order to make sure they are Ig gene amplifications and the sequences of the primers and the tags are intact. DNA concentration of PCR products from each sample were determined by PicoGreen dye and fluorospectrometer (Nanodrop). According to these results, a mixture containing 10^9^ molecules of PCR products from each sample was prepared and sent to sequencing. HTS was performed using the 454 GS FLX Titanium platform by DYN Diagnostics Ltd., the sole representative in Israel of Roche Diagnostics. Raw data files containing a total of ~120,000 reads were received when the HTS was completed. Raw data files can be downloaded from the SRA database, accession number PRJNA206548 (Runs: SRR873440, SRR873441, SRR873442).

### HTS raw data pre-processing

To process the 454 raw data, we used our program Ig-HTS-cleaner (73). Ig-HTS-cleaner discards artifact sequences, assigns the sequences to samples according to their MID tags, identifies primers, and discards sequences much shorter or longer than the expected length of an Ig variable region gene, or sequences with average quality scores below a defined threshold. Parameters used in the Ig-HTS-Cleaner run were as follows. Average quality score threshold of 20, a maximum of 2 allowed mismatches in the primer search, 75% of the primer’s length to search, and a range of 25 bases at the ends of the read for the MID and primers search (Table S3 in Supplementary Material).

Next, we discarded duplicate sequences, which are completely identical sequences, from each sample. We cannot exclude the possibility that duplicate sequences are a result of the PCR amplification; hence the existence of many identical sequences in a sample does not necessarily indicate that the sequence is found in the original biopsy in the same frequency.

Afterward, we used our program Ig-Indel-Identifier (Ig Insertion – Deletion Identifier) (73), in order to identify legitimate and artifact insertions and/or deletions (indels) in the sequences. Parameters used in the Ig-Indel-Identifier run were as follows. HPT length was set to 2, quality score threshold (for suspected point mutations) of 25, and the number of sequences in the same clone containing the same indel was set to 1 (Table S4 in Supplementary Material). Table 4 presents the numbers of unique sequences from each condition after discarding sequences with suspected indels. These were the final numbers of sequences that were analyzed.

**Table 4:** **Number of unique sequences^a^, without suspected indels^b^, from each condition**.

	CLN	GNHP	GHP	MALT-L	DLBCL	Total
Number of patients (samples)	19	7	3	3	5	37
Number of unique sequences	23,308	4,676	3,373	3,851	4,389	39,597
Range of sequences^c^	384–3,353	75–1,105	513–1,406	838–1,854	267–1,601	
Dominant clone sizes^d^				360 (249)	162 (49)	
				461 (399)	325 (257)	
				408 (321)	58 (42)	
					78 (47)	
					418 (193)	

*^a^Unique sequence: a sequence that differs from all other sequences due to one or more insertion(s), deletion(s), or point mutation(s)*.

*^b^After sequences with suspected indels were discarded*.

*^c^The lowest-to-highest numbers of unique sequences without suspected indels in each sample from each condition*.

*^d^The number of unique sequences in the dominant clone, and the number of the second dominant clone (in parentheses), in the lymphoma samples. Each couple of numbers represents one-sample*.

### Germline VDJ segment identification and assignment into clones

Clonally related sequences were identified by identical V(D)J segments and by highly homologous sequences of the CDR3 of their Ig genes. For gene segment identification, we used SoDA (75). We computationally grouped the sequences into clones based only on their V, D, and J segments. We aligned clonally related sequences using ClustalW2 (76), in order to confirm that the CDR3 in the clonally related sequences were highly homologous. If not, we separated the sequences into clonally related groups according to the different CDR3 sequences.

### Repertoire analysis

We enumerated the clones based on V(D)J combinations. Results are presented as the average sample percentages of clones of each VH–JH combination, across all samples within the same group. Using the percentages normalizes for different numbers of sequences and/or clones, due to sampling of different numbers of B-cells or obtaining different DNA quantities in each case.

In order to examine the relationships between the VDJ combinations used in each repertoire, we needed to compare the observed repertoires to repertoires predicted under some model, for example, under the assumption that the expression of each gene in each VDJ combination (e.g., V1D1J1) is independent of that of other genes. Immunologists call this assumption “the product rule” (77). Deviations from this assumption can thus point at interdependencies between the V, D, and J genes. We decided to look only at the gene family level, as higher resolution (genes, alleles) would give extremely large numbers of possible combinations, far from the number of combinations observed and therefore the frequencies of each expected combination at the gene or allele level would be close to zero. Thus, each observed gene combination would be significantly different from the expected. Using only families of the VDJ segments would solve this problem. For each sample, we counted the number of unique sequences that used each V, D, or J family. We then calculated the frequency of each V/D/J family as the number of unique sequences using this family divided by the total number of unique sequences in the sample. We then created all possible combinations that can be made using the observed VDJ families, and defined their expected frequencies as the product of the V/D/J family frequencies calculated in the previous step. Next, we calculated the actual frequencies of the observed combinations (number of unique sequences in each observed combination divided by the total number of unique sequences). There was no point in creating combinations with families that did not appear in the sample, as there was no meaning of calculating frequencies of non-existing combinations.

In order to know whether a combination was expressed more or less than expected, we calculated the expression: log_2_(observed/expected). If a specific combination was over-expressed compared to the expected frequency, the ratio inside the logarithm would be larger than 1, as the observed frequency would be larger than the expected, and thus the logarithm would be positive. On the other hand, if a specific combination was under-expressed compared to the expected frequency, the ratio inside the logarithm would be smaller than 1, and thus the logarithm will be negative. Combinations that were not observed at all received the value (−∞), because the expression inside the logarithm was zero. This step was repeated for each sample. It is important to note that most of the combinations (>80%) were observed in a significantly different frequency than expected. Out of these combinations, 99% were under-represented (because the number of combinations observed is smaller than the potential number), and only 1% of the combinations were over-expressed compared to the expected frequency, and any such case of over-expression was thus particularly noticeable.

The final step was to unite all combinations from all samples as follows: we created a matrix, where rows represented samples and columns represented VDJ combinations. For each combination and for each sample, we inserted the logarithm that was calculated as above. If a sample did not have a specific combination, the cell would be left unfilled. The full matrix was used to carry out the statistical tests. In order to examine whether some combinations tend to appear more or less than expected, we carried out a one-sample *t*-test on each of the conditions. In order to examine differences in combination usage between conditions, we carried out a two-sample ANOVA test.

In order to graphically present repertoires, we only plotted V–J repertoires, not showing the DH segments used in each VH–JH combination. There are several ways of presenting also the DH genes used in the repertoires (78, 79). However, as mentioned above, DH segments are sometimes misidentified, so we preferred to focus on V and J segments.

### Diversity analysis

#### Clones in samples can be regarded as species in habitats

In the case of lymphocyte clonal repertoire samples (e.g., those obtained from tissue biopsies), we treat each sample as a sample from a habitat, in which the “species” are the BCR or TCR clones found in the sample. Each of the clones may be composed of a number of different sequences. In TCR clones, all sequences are identical, but in BCR clones sequences from the same clone may be different due to SHM, and one may choose to use only unique sequences found, or all sequences including multiplicate ones. The latter choice depends on whether identical sequences coming from different cells can be identified as such, or cannot be distinguished from sequence duplications caused by PCR amplification. If the former is true (as when using random barcoding in the PCR primers), then the number of sequences that come from different cells is known, and can be used to estimate diversity. If not, then TCR diversity cannot be estimated, and BCR diversity can only be estimated based on the numbers of unique sequences and thus would usually only gives a minimum estimate of the total diversity, as we have done in this study.

#### Diversity indices

In order to quantify the diversity of clonal repertoires (such as antibody/BCR or TCR gene repertoires) in each experimental or clinical condition, and later to be able to compare between two or more conditions, we used diversity indices (such as the Species Richness, the Shannon entropy, or the Simpson concentration, which are indices of order 0, 1, and 2, respectively) (80). These indices take into account the number of species and (in indices of order >0) the frequency of members of a species (in our case, sequences) of each species (in our case, clone) in each habitat sample. In indices of order 0, diversity is defined simply as number of species (in our case, lymphocyte clones) in a sample. In order 1 indices, clone size (or frequency) is taken into account, as in the Shannon entropy when diversity is the sum of $\left[-p_{\text{i}}^{*}\ln\left(p_{\text{i}}\right)\right]$, where *i* represents a species or clone and *p*_i_ represents its size (the number of members/sequences, see below). Order 2 indices attribute more weight to large clones, as in the Simpson concentration, which is the sum of p_i_^2^. In our studies, we used both order 1 and 2 diversity indices, i.e., the Shannon entropy and the Simpson concentration.

#### Diversity measures

From the sample diversity indices, we have calculated the alpha, beta, and gamma diversity measures for each condition (80). The alpha diversity measure represents the average sample diversity in each condition/population. In order to calculate alpha, we calculate the alpha diversity of each sample, and then average over all samples from the same condition. The gamma diversity measure represents the “global” repertoire diversity across all samples studied in each condition/population. It is calculated as the diversity of the pool containing all the sequences from all the samples from the same condition/population.

Finally, the beta diversity measure, which represents the diversity component resulting from the variability between samples, is derived from the alpha and gamma measures using the method of Jost et al. (80). The beta diversity measure is calculated as the gamma diversity of each condition/population divided by the alpha diversity (average of the diversities of individual samples). In order to allow an intuitive comparison between the diversities of each of the groups, all the diversity measures can be expressed as their number equivalents (80), which reflect the number of equally sized clones needed to produce the given value of the diversity index.

#### Estimating the full repertoire from the sample

Considering the large number of sequences that were observed only once in each sample, it is likely that many rare clones in an individual’s original full repertoire were not detected. To account for the presence of unobserved “species” (clones), all diversity measures can be estimated for whole repertoires (rather than calculated for the sample) using the method described by Chao and Shen (81), which is based on a non-parametric estimation of diversity indices where there are undetected species. Chao and Shen’s approach utilizes the concept of sample coverage to adjust the diversity indices for clones that escaped sampling. The sample coverage is estimated from the proportion of species/sequences that are observed only once within a sample.

**Figure 5:**
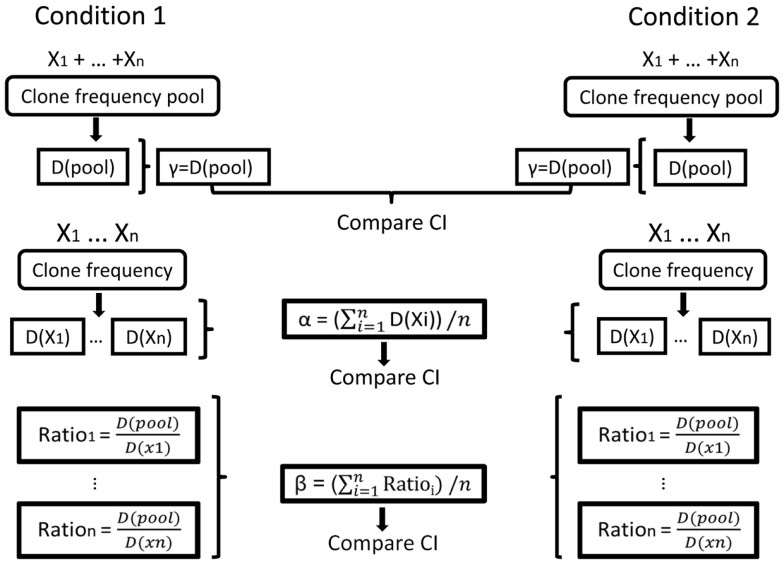
**Illustration of the calculation of repertoire diversity**. First, the diversity indices (Shannon entropy, Simpson concentration index, etc.) are calculated for the pool of samples in each condition and also for each sample separately (samples are denoted by *X*_1_, …, *X*_n_. Diversity indices are denoted by D (sample) or D (pool)). Next, the distribution measures (α, β, γ) are calculated for each condition using the samples. γ is the pool diversity, α is the mean sample diversity, and β for each sample is γ divided by D (sample). Finally, the CIs of the distribution measures are compared between the populations/conditions.

In our Ig gene repertoire studies, the abundance data (numbers of unique sequences) of antibody clones in each sample were used to estimate the mean, standard error, and 95% CI of the total number of unique sequences in clones within each sample. This was done using SPADE©, a program designed for diversity calculations (81). The alpha diversity for each sample, and the gamma diversities for combined samples in each condition, were then calculated from the order 1 or 2 diversity indices of the estimated total repertoires, also using SPADE©(81). In principle, beta is calculated as the average alpha of all samples in the condition divided by the gamma of the condition, as explained above. In order to compare between conditions, however, we needed to calculate CI for beta. This was done by calculating beta index per sample (alpha of the sample divided by the gamma of the condition) and then calculating the CI for each condition (Figure 5).

### Similarity analysis

Another method we used to compare between conditions is the Morisita similarity index (82). SPADE©(81) was used to calculate a similarity matrix, in which we measured each individual repertoire’s similarity to all other individual repertoires. The average of similarity indices of individuals in a given group to those in another group represents the similarity index for the comparison between the two groups. A value close to 1 represents high similarity between two groups, and a value close to 0 represents low similarity.

The highest values of the Morisita similarity indices representing the highest similarity were rather low and relatively far from 1, indicating the sensitivity of this method. However, they were consistent with observed repertoire diversities.

### Ig lineage tree analyses

Clonally related Ig gene sequences from each sample were used to create mutational lineage trees using our program IgTree©(83), as described in previous work (29, 30). All trees were measured using our program MTree©, quantifying the graphical properties of the trees (84, 85). A thorough statistical analysis has concluded that seven specific tree characteristics possess the highest correlation values with the biological parameters and are hence most informative (67). As described there, these properties are the minimum root to leaf path length, the average distance from a leaf to the first split node/fork, the average outgoing degree, that is the average number of branches coming out of any node, the root’s outgoing degree, minimum distance between adjacent split nodes/forks, the length of the tree’s trunk and minimum distance from the root to any split node/fork. The analysis in this study has thus focused on these properties. Comparison between lineage tree characteristics of different conditions was done using the non-parametric Mann–Whitney *U*-test, as normal distributions (required by tests such as Student’s *t*-test) could not be assumed. We used the FDR correction (86) for multiple comparisons.

Replacement (R) and silent (S) mutation analysis methods attempt to measure the extent of selection operating on the diversifying clones. These methods compare the frequencies of replacement mutations found in the frame-work and CDR regions of mutated Ig gene sequences to their expected frequency, based on codon usage of the germline sequence. We used the updated focused binomial test by Hershberg et al. (87, 88). The numbers of observed mutations were pooled for each data group by IgTree©, and the new focused binomial formula (88) was calculated using Microsoft Excel©. This measure was also performed for each sample separately, yielding the same results; however, when comparing conditions, we chose to show the pooled analysis for simplicity. Additional mutational analyses were carried out as described in previous studies (27, 28, 30), however, no significant differences between the conditions were found.

## Author Contributions

Miri Michaeli performed all steps from DNA extraction from samples to bioinformatical analysis of the sequences, and wrote the manuscript. Hilla Tabibian-Keissar supervised the molecular process. Ginette Schiby revised the samples to confirm the diagnosis. Gitit Shahaf and Yishai Pickman developed the diversity analysis. Lena Hazanov developed the bioinformatical analyses. Kinneret Rosenblatt was in charge of the laboratory in which the molecular work was performed. Deborah K. Dunn-Walters advised the author throughout the study. Ramit Mehr and Iris Barshack supervised the molecular work and the analyses performed, and finalized the manuscript. All authors read and approved the final manuscript.

## Conflict of Interest Statement

The authors declare that the research was conducted in the absence of any commercial or financial relationships that could be construed as a potential conflict of interest.

## Supplementary Material

The Supplementary Material for this article can be found online at http://www.frontiersin.org/Journal/10.3389/fimmu.2014.00264/abstract

## Abbreviations

AML, acute myeloid leukemia; B-CLL, chronic lymphocytic leukemia; BCR, B-cell receptor; CDR, complementary determining region; CI, confidence intervals; CLN, control lymph node; DLBCL, diffuse large B-cell lymphoma; FL, follicular lymphoma; GHP, gastritis with *H. pylori* background; GNHP, gastritis without *H. pylori* background; *H. pylori*, *Helicobacter pylori*; HTS, high-throughput sequencing; Indels, insertions and/or deletions; MALT, mucosa-associated lymphoid tissue; MALT-L, MALT lymphoma; MID, molecular identification; MM, multiple myeloma; SHM, somatic hypermutation.
