# Supplementary material for: Immunoglobulin Gene Repertoire Diversification and Selection in the Stomach – From Gastritis to Gastric Lymphomas
Source: Front Immunol. 2014 Jun 3;5:264. doi: 10.3389/fimmu.2014.00264 (PMC4042156; doi:10.3389/fimmu.2014.00264)
Supplement: Supplementary file 1 [file DataSheet_1.DOCX]

**Online supplemental material**

**
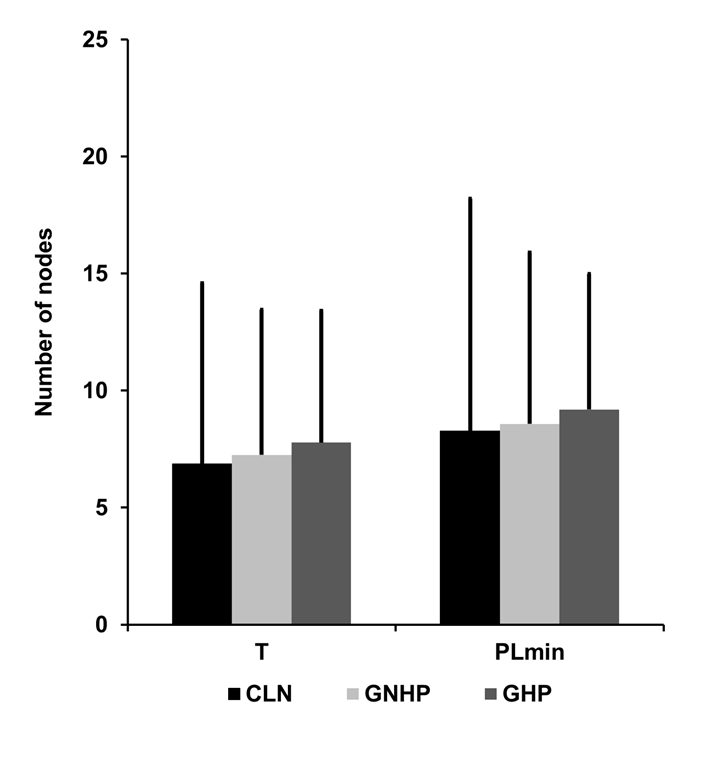
**

**Figure S1.** Lineage tree analysis – comparison between clones from the 19 CLN samples (1682 trees), the seven GNHP samples (282 trees) and the three GHP samples (212 trees).

**
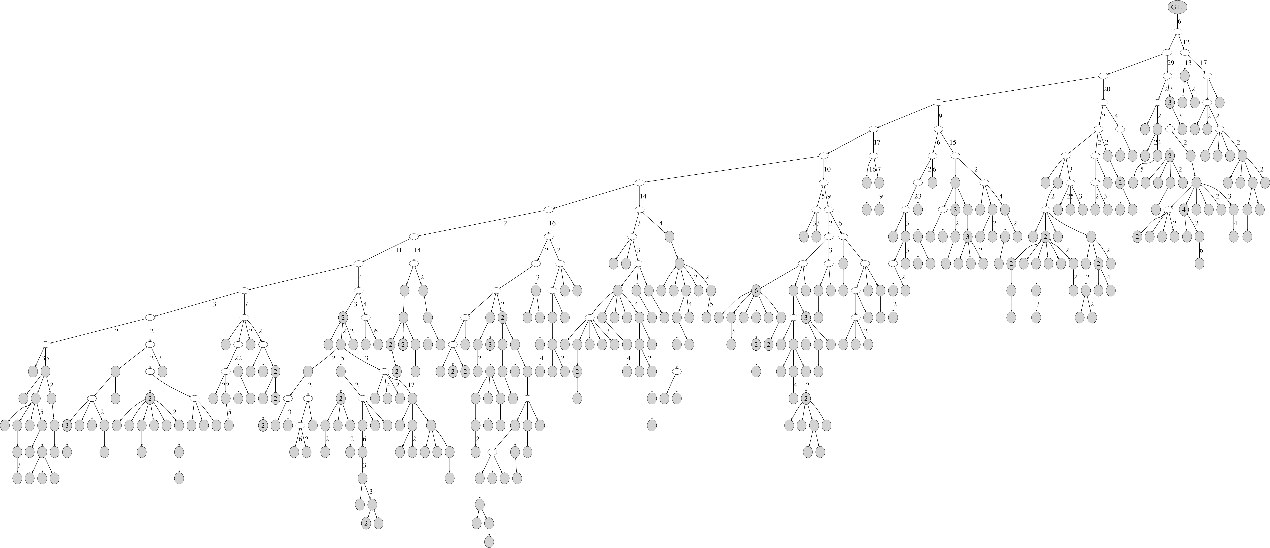
Figure S2**. A representative example of a dominant MALT-L tree.


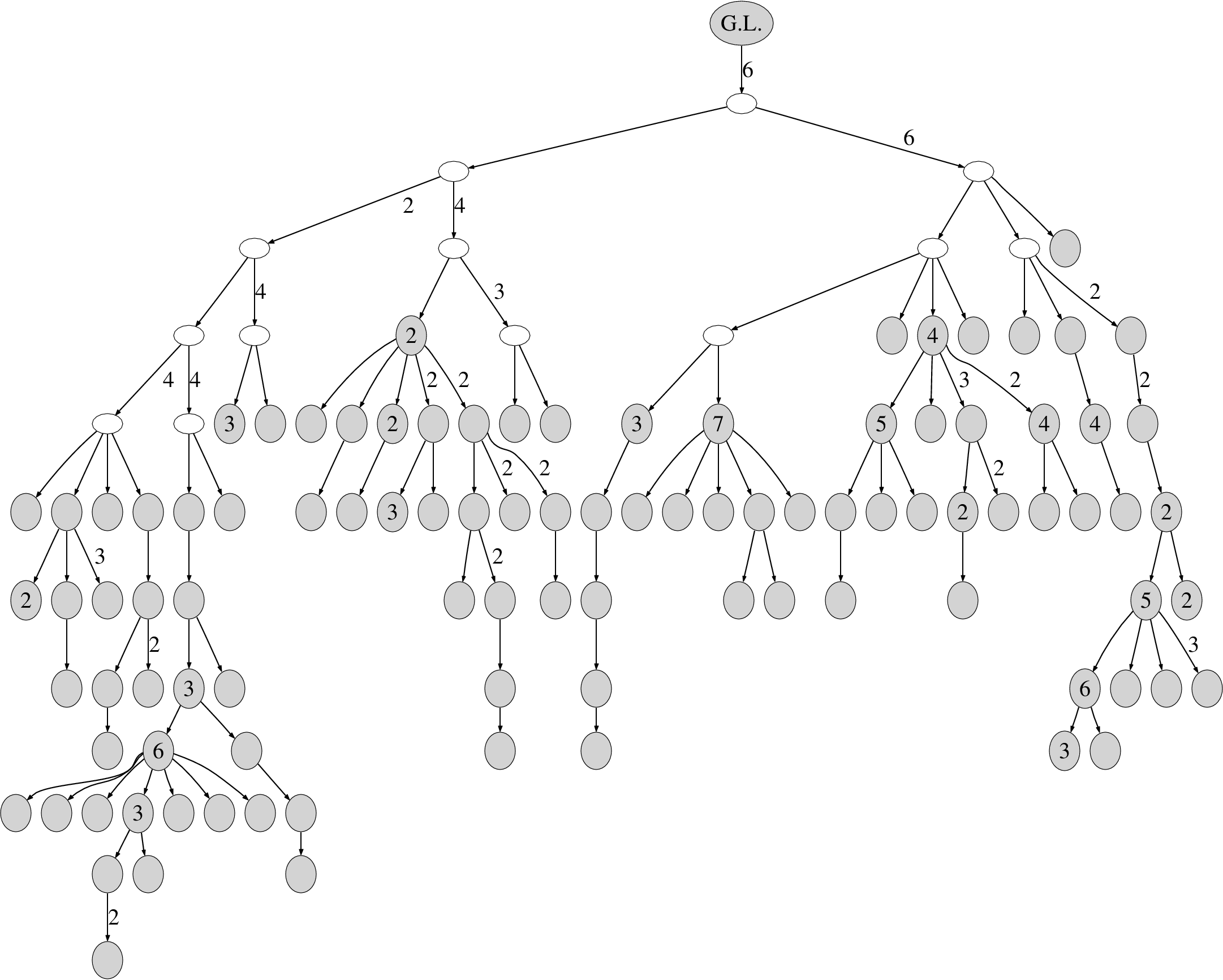


**Figure S3**. A representative example of a dominant DLBCL tree.

**Table S1: Patient samples analyzed in this study.**

| **Source** | **Sample number** | **Patient age** | **Gender** | **Description** |
| --- | --- | --- | --- | --- |
| LN | 1 | 18 | M | Nodular lymphocytic. Behind right ear, biopsy: RLN, polykaryocytes are present. Possible viral infection. |
| LN | 2 | 20 | M | Neck, RT. posterior triangle: RLN. |
| LN | 3 | 20 | M | Cervical RLN showing follicular and paracortical hyperplasia. |
| LN | 4 | 20 | F | Cervical LN, Reactive follicular hyperplasia. NEM. |
| LN | 5 | 27 | F | Cervical RLN. NEM. |
| LN | 6 | 27 | F | LT. Axillary dissection, RLN. NEM. |
| LN | 7 | 33 | F | Cervical LN, Florid reactive follicular hyperplasia. NEM. |
| LN | 8 | 44 | F | LT. Axillary LN, Axillary lymphadenopathy. |
| LN | 9 | 48 | M | RT. Inguinal LN, lymphadenopathy. |
| LN | 10 | 54 | F | RT. Axillary LN, Axillary lymphadenopathy. |
| LN | 11 | 63 | M | LT. Axillary LN, fever of unknown origin, lymphadenopathy. |
| LN | 12 | 66 | M | RT. and LT. Pretracheal LNs, and carina, with reactive change and sinus histiocytosis. |
| LN | 13 | 69 | M | RT. pre tracheal LNs, A conglomerates of RLN with sinus histiocytosis. |
| LN | 14 | 70 | M | LT. Inguinal LN, RLN showing severe congestion, fatty infiltration and fibrosis. NEM. |
| LN | 15 | 70 | F | Lingual tonsil, biopsy: Lymphoid tissue covered by mildly hyperplastic squamous epithelium. Mild chronic and acute inflammation is present. NEM. |
| LN | 16 | 73 | F | Cervical LN, Follicular and interfollicular hyperplasia. The interfollicular lymphocytes are small and very uniform. |
| LN | 17 | 77 | F | Pelvic RLN. NEM. |
| LN | 18 | 78 | F | Suspected lymphoma. RT inguinal LN,  Two RLN with fat infiltration. Adipose tissue with a few aggregates of foamy cells probably the result of fat necrosis. |
| LN | 19 | 78 | M | Central of bleeding. RT tonsil, tonsillectomy: Tonsillar lymphoid area with acute inflammation and surrounding soft tissue. |
| GHP | 1 | 25 | F | Acute and chronic gastritis- equivocal presence of *H. pylori*. |
| GHP | 2 | 66 | F | Chronic active gastritis with numerous plasma cells *H. pylori* positive. |
| GHP | 3 | 68 | M | CAG with *H. pylori* and intestinal metaplasia. |
| GNHP | 1 | 30 | M | Normal gastric mucosa, mild chronic gastritis. |
| GNHP | 2 | 36 | F | Hiatus hernia. |
| GNHP | 3 | 50 | F | Gastric ulcer, CAG. |
| GNHP | 4 | 52 | F | Gastric polyps. Focal acute and chronic gastritis and intestinal metaplasia. |
| GNHP | 5 | 65 | M | Nodular gastric mucosa. Chronic gastritis and intestinal metaplasia. |
| GNHP | 6 | 69 | M | Gastric erosions. Mild chronic inflammation, small intestinal metaplasia. |
| GNHP | 7 | 76 | M | Focal gastric nodule. Mild chronic gastritis and lymphoid hyperplasia. |
| MALT-L | 1 | 67 | F | Malignant lymphoma |
| MALT-L | 2 | 68 | M | S/P treatment for MALT-L- gastric erosion. Histology: lymphoma. |
| MALT-L | 3 | 75 | M | Stomach lymphoma involving the mucosa and submucosa. The surface epithelium is ulcerated. Histology: MALT-L. |
| DLBCL | 1 | 34 | M | Partial gastrectomy – lymphoma, large cell type, and the tumor is limited to the mucosa. |
| DLBCL | 2 | 47 | F | Ulcerated tumor composed by round cells invading the muscular coats and reaching the serosa. The histologic pictures favor a large cell lymphoma. |
| DLBCL | 3 | 61 | F | Stomach localized large cell lymphoma with ulceration and associated severe chronic and acute inflammation, fibrosis and peritonitis. |
| DLBCL | 4 | 75 | M | Stomach (partial gastrectomy) large B cell lymphoma with follicular areas involving the mucosa and submucosa. The surface epithelium is ulcerated. Surgical edges are free of tumor. |
| DLBCL | 5 | 89 | F | Biopsy from gastric mass smooth muscle infiltrated by a predominantly large B cell lymphoma with numerous mitoses. Profile rating cell nuclear antigen is positive in 80-90% of the tumor cells. |

**Table S1.** LN: lymph node, used as control; RLN: reactive lymph node; RT: right; LT: left; NEM: no evidence for malignancy; CAG: chronic active gastritis.

**Table S2: VDJ combinations with differences in expression between conditions**.

| Combination | Condition | N | Mean deviation | Std. Deviation | Lower bound 95% Confidence | Upper bound 95% Confidence | p-value [One-Sample T Test (Test Value=0)] | p-value (ANOVA) |
| --- | --- | --- | --- | --- | --- | --- | --- | --- |
| V1D1J6 | GNHP | 7 | -16.45 | 17.826 | -26.86 | -6.05 | .002 | 0.031 |
|  | DLBCL | 5 | 1.29 | .638 | .50 | 2.09 | .011 |  |
| V1D1J2 | CLN | 4 | -38.96 | .639 | -40.35 | -37.58 | 2.3E-17 | 7.42E-09 |
|  | DLBCL | 4 | .48 | .174 | -.07 | 1.04 | .069 |  |
| V1D1J2 | GNHP | 4 | -35.63 | .75 | -38.03 | -33.24 | 2.07E-05 | 1.36E-06 |
|  | DLBCL | 4 | .48 | .174 | -.07 | 1.04 | .069 |  |
| V1D1J2 | GHP | 2 | -36.88 | 1.02 | -49.86 | -23.9 | .018 | 1.74E-05 |
|  | DLBCL | 4 | .48 | .174 | -.07 | 1.04 | .069 |  |
| V2D2J1 | CLN | 12 | -30.56 | 3.18 | -37.56 | -23.56 | 1.1E-06 | 2.63E-06 |
|  | MALT-L | 2 | 2.26 | .95 | -9.87 | 14.38 | .255 |  |
| V2D2J1 | GNHP | 3 | -30.01 | .718 | -33.11 | -26.92 | .001 | 2E-06 |
|  | MALT-L | 2 | 2.26 | .95 | -9.87 | 14.38 | .255 |  |
| V2D2J1 | GHP | 3 | -29.89 | 1.90 | -38.05 | -21.72 | .004 | 2.06E-06 |
|  | MALT-L | 2 | 2.26 | .95 | -9.87 | 14.38 | .255 |  |
| V2D2J1 | DLBCL | 3 | -32.99 | 1.32 | -38.69 | -27.29 | .002 | 1E-06 |
|  | MALT-L | 2 | 2.26 | .95 | -9.87 | 14.38 | .255 |  |
| V3D3J3 | GNHP | 6 | -39.6 | 1.64 | -41.32 | -37.88 | 2.6E-08 | 0.025 |
|  | MALT-L | 3 | 3.77 | 3.077 | -3.87 | 11.42 | .168 |  |
| V3D3J5 | GNHP | 5 | -40.55 | 1.265 | -42.12 | -38.97 | 2.27E-07 | 0.01 |
|  | GHP | 3 | 4.00 | 5.000 | -8.42 | 16.43 | .300 |  |
| V5D6J4 | CLN | 18 | -30.50 | 21.29 | -41.09 | -19.92 | 1.2E-05 | .025 |
|  | DLBCL | 5 | .93 | 1.97 | -1.51 | 3.38 | .349 |  |

**Table S2.** Descriptive statistics of the mean deviation [log_2_(observed/expected)] for each V(D)J combination in each condition. The column "N" represents the number of samples in each condition that contained the specific VDJ combination. The rightmost column represents the p-value of the two-sample ANOVA test. The column "p-value [One-Sample T Test (Test Value=0)]" represents the statistical significance of the difference of between the observed and the expected frequencies, based on the null hypothesis that the ratio of the [observed/expected] frequencies is zero.

**Table S3: A summary of Ig-HTS-Cleaner results.**

| No. of sequences with tags | No. of sequences without primers | No. of sequences that failed due to length | No. of sequences that failed in quality check | No. of remaining sequences | No. of unique sequences |
| --- | --- | --- | --- | --- | --- |
| 112,490 | 4,494 | 34 | 714 | 107,248 | 48,371 |

**Table S3**. Parameters used in the Ig-HTS-Cleaner run were as follows. Average quality score threshold of 20, a maximum of 2 allowed mismatches in the primer search, 75% of the primer's length to search, and a range of 25 bases at the ends of the read for the MID and primers search.

**Table S4: A summary of Ig-Indel-Identifier results.**

| Total | No. of sequences w/o indels | Total no. of sequences with indels | % of sequences with indels | No. of uncertain indels^a^ | No. of sequences with artifact indels^b^ |
| --- | --- | --- | --- | --- | --- |
| 48,371 | 39,597 | 8,774 | 22.1 | 153 | 8,617 |

**Table S4**. Parameters used in the Ig-Indel-Identifier run were as follows. HPT minimum length was set to 2, and the minimum number of additional sequences in the same clone containing the same indel (that are required to confirm an indel) was set to 1.

**Table S5: Distributions of the numbers of sequences in tree nodes, in the dominant and the second dominant clones of lymphoma samples.**

| Sample | The dominant clone | | The second dominant clone | |
| --- | --- | --- | --- | --- |
|  | Number of sequences in a node | number of nodes | Number of sequences in a node | number of nodes |
| MALT1 | 1 | 211 | 1 | 129 |
|  | 2 | 36 | 2 | 20 |
|  | 3 | 15 | 3 | 10 |
|  | 4 | 9 | 4 | 1 |
|  | 5 | 4 | 5 | 2 |
|  | 6 | 1 | 6 | 3 |
|  |  |  | 9 | 1 |
|  |  |  | 10 | 1 |
| MALT2 | 1 | 238 | 1 | 368 |
|  | 2 | 29 | 2 | 23 |
|  | 3 | 11 | 3 | 6 |
|  | 4 | 5 | 4 | 1 |
|  | 5 | 4 | 6 | 1 |
|  | 6 | 2 |  |  |
|  | 7 | 1 |  |  |
|  | 8 | 2 |  |  |
|  | 9 | 1 |  |  |
|  | 10 | 1 |  |  |
|  | 11 | 1 |  |  |
|  | 13 | 1 |  |  |
|  | 14 | 1 |  |  |
| MALT3 | 1 | 112 | 1 | 157 |
|  | 2 | 10 | 2 | 13 |
|  | 3 | 5 | 3 | 6 |
|  | 4 | 4 | 4 | 4 |
|  | 5 | 2 | 5 | 4 |
|  | 6 | 4 | 6 | 2 |
|  | 7 | 1 | 8 | 2 |
|  | 8 | 1 | 15 | 1 |
|  | 9 | 1 | 18 | 1 |
|  | 11 | 2 | 23 | 1 |
|  | 12 | 2 |  |  |
|  | 13 | 1 |  |  |
|  | 14 | 1 |  |  |
|  | 17 | 1 |  |  |
|  | 25 | 1 |  |  |
|  | 26 | 1 |  |  |
|  | 46 | 1 |  |  |
| DLBCL1 | 1 | 30 | 1 | 32 |
|  | 2 | 9 | 2 | 5 |
|  | 3 | 2 |  |  |
|  | 4 | 1 |  |  |
| DLBCL2 | 1 | 40 | 1 | 45 |
|  | 2 | 5 | 2 | 1 |
|  | 3 | 2 |  |  |
|  | 4 | 1 |  |  |
|  | 5 | 1 |  |  |
|  | 6 | 1 |  |  |
|  | 7 | 1 |  |  |
| DLBCL3 | 1 | 72 | 1 | 34 |
|  | 2 | 7 | 2 | 6 |
|  | 3 | 7 | 3 | 1 |
|  | 4 | 2 |  |  |
|  | 5 | 7 |  |  |
|  | 6 | 2 |  |  |
| DLBCL4 | 1 | 184 | 1 | 91 |
|  | 2 | 13 | 2 | 16 |
|  | 3 | 14 | 3 | 5 |
|  | 4 | 5 | 4 | 3 |
|  | 5 | 2 | 5 | 1 |
|  | 6 | 3 | 6 | 2 |
|  | 7 | 1 | 7 | 5 |
|  | 8 | 1 | 8 | 1 |
|  | 10 | 1 | 9 | 2 |
|  |  |  | 14 | 1 |
|  |  |  | 15 | 1 |
| DLBCL5 | 1 | 174 | 1 | 81 |
|  | 2 | 27 | 2 | 9 |
|  | 3 | 13 | 3 | 6 |
|  | 4 | 9 | 4 | 2 |
|  | 5 | 3 | 5 | 1 |
|  | 6 | 6 | 6 | 1 |
|  | 7 | 2 | 8 | 1 |
|  | 8 | 2 | 9 | 3 |
|  | 10 | 2 | 11 | 2 |
|  | 14 | 1 |  |  |
|  |  |  |  |  |
|  |  |  |  |  |
